# Happy feet: the key roles of podosomes and invadopodia in trophoblast invasion at the maternal-fetal interface

**DOI:** 10.3389/fendo.2025.1576732

**Published:** 2025-05-30

**Authors:** Padma Murthi, Emily Overton, Shaun P. Brennecke, Rosemary J. Keogh

**Affiliations:** ^1^ Department of Maternal Fetal Medicine, Pregnancy Research Centre, Royal Women’s Hospital, Parkville, Australia; ^2^ Department of Obstetrics, Gynaecology and Newborn Health, University of Melbourne, Parkville, Australia

**Keywords:** cell invasion, cell migration, invasive projection, podosome, invadopodia, cancer, pregnancy, trophoblast cell

## Abstract

Cells move by forming specialized projections or invasive feet known as podosomes in normal invasive cells and invadopodia in transformed and cancer cells. An understanding of invasive projections of trophoblasts at the maternal-fetal interface and their formation is important for developing novel therapies for pregnancy complications where invasion is abnormal, in instances where over- or under-invasion of cells manifests as serious pregnancy pathologies such as accreta or preeclampsia. Podosomes and invadopodia have distinctive morphological and molecular features that are used to distinguish them from each other. Despite this, there is still debate and uncertainty around how to definitively classify them. Analyses of novel models of cell invasion have demonstrated the existence of hybrid structures that are neither true podosomes nor invadopodia but which display features of both. This raises the question as to whether the classification of invasive structures needs redefining.

## Introduction

In order for cells to invade into tissue they must interact with and digest the extracellular matrix. This normal physiological process can lead to serious disease pathologies when it is defective with cancer being the best known and researched example. Understanding cell invasion is thus critically important to gain insights for developing treatments for diseases where it is abnormal. Invasive cells form specialized projections which make physical contact with the extracellular matrix and secrete proteases, thus enabling them to adhere, migrate and invade. Known as podosomes in non-transformed cells and invadopodia in transformed and cancer cells, these invasive projections each have distinctive characteristics (1–4). Despite a growing body of knowledge on podosomes and invadopodia, there are still many unknowns. There is much debate about whether they are different structures, whether one is a precursor of the other or whether they are merely two representations of the same structure altered by the *in vivo* environment where it forms (5–9). In order to address this, it is important to study novel models of cell invasion. This will help to define if podosomes and invadopodia are two extremes of a continuum of structures or if they are of independent origin and will help to refine the current theories on how invasive projections are classified. These vitally important insights will impact current studies targeting the control of cell invasion as a therapeutic strategy.

## Podosomes and invadopodia

### Morphological *features*

**Figure 1:**
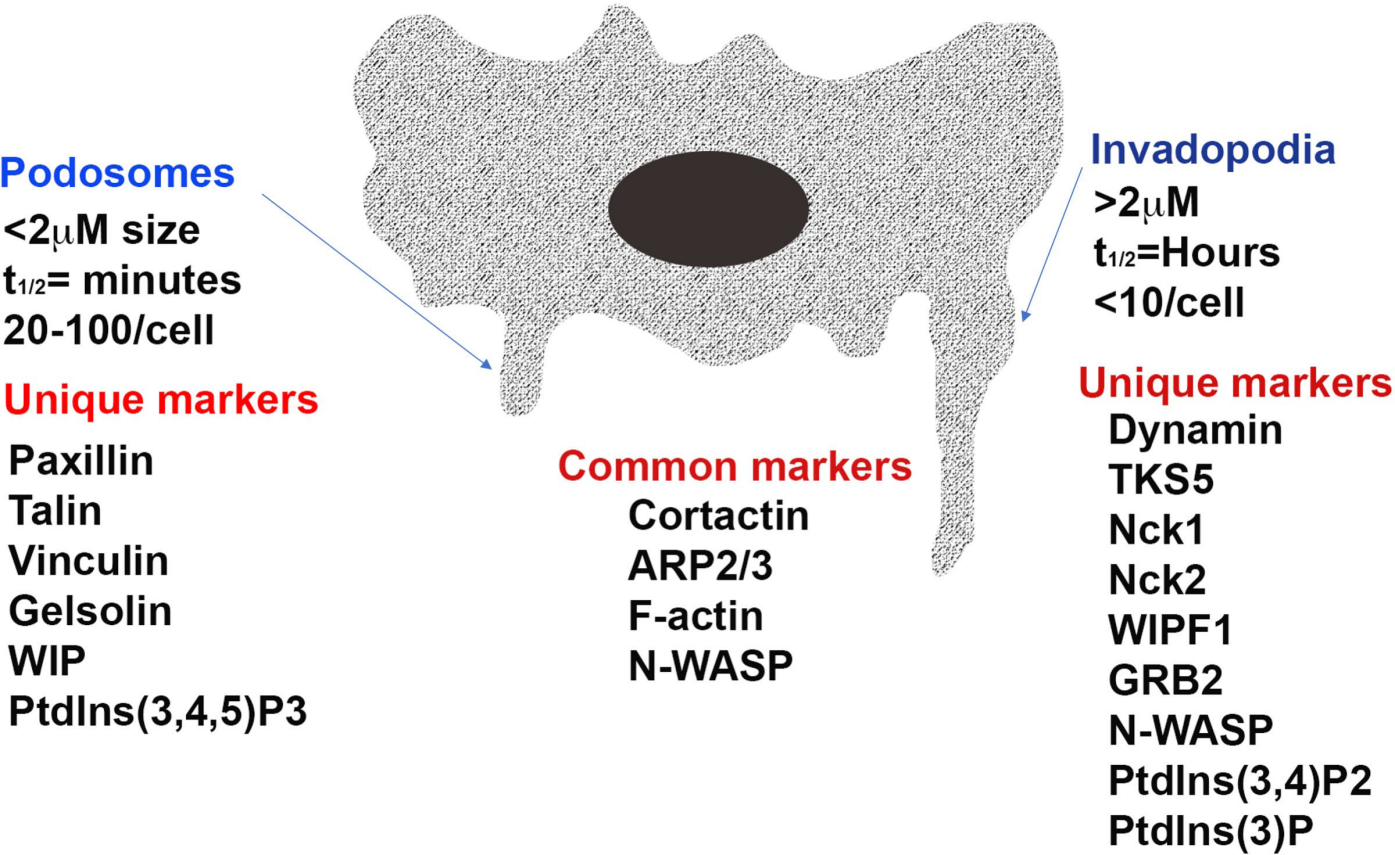
The structure of the invasive projections of a cell. Distinct and common features of a podosome and invadopodia of an invasive cell is depicted in Image 1. As shown the structure of invasive projections of podosomes and invadopodia are characterized by high actin polymerization activity and short, disorganized actin cores surrounded by a ring of adhesion structures. While the podosome cores are dynamic and have a turnover rate of minutes, invadopodia can remain stable for several hours. Invadopodia also extend into and invade the extracellular matrix to a greater depth through aggressive matrix degradation. ARP2/3, actin related protein 2/3 complex; N-WASP, Neural Wiskott-Aldrich syndrome protein; WIPF1, WASP-interacting protein; TKS5 tyrosine kinase substrate with five SH3 domains; Nck, non-catalytic region of tyrosine kinase; GRB2, growth factor receptor-bound protein 2; PtdIns(3,4,5)P3, phosphatidylinositol-3,4,5-triphosphate; PtdIns(3,4)P2, phosphatidylinositol-3,4-bisphosphate; PtdIns(3)P, phosphatidylinositol-3-phosphate.

Cells make contact with their surroundings via several types of specialized structures which enable them to either adhere to, or move through, the extracellular environment (10). Focal adhesions and focal contacts are points of adherence which attach cells to the substratum and can act as mechanosenors (11–13). They anchor stable cross-linked bundles of actin filaments at sites of integrin clustering. In order for cells to be able to move and invade they must form structures that can be rapidly assembled and dis-assembled and which can digest the extracellular matrix. For this purpose, actin-rich membrane protrusions known as podosomes and invadopodia are formed. These invasive feet extend from cells and are associated with sites where there is digestion of the extracellular matrix by proteases secreted by the cells. The similarities and differences between these two structures are the subject of much discussion although there are morphological and molecular features that distinguish them (See **Figure 1**) including their length, duration of lifespan, number per cell and the proteins and lipids present in and secreted by the projections (1, 2, 12–14). Functionally it has been suggested that podosomes act to promote directed cell movement while invadopodia primarily function to digest extracellular matrix (15–17).

Podosomes are formed by normal invasive cells including monocytes, osteoclasts, endothelial cells and vascular smooth muscle cells and digest matrix via mechanisms involving membrane type-matrix metalloproteinase (MT1-MMP) and the urokinase receptor uPAR (6, 18, 19). They are typically less than 2 µm in length, have a lifespan measured in minutes and 20–100 form per cell (1, 2). Podosome adhesions are dot-like in nature and have a core of actin and associated proteins embedded in a ring of adhesion plaque proteins including paxillin, talin or vinculin (1, 6). Invadopodia is the name given to invasive projections formed by cancerous and transformed cells (2). They use matrix metalloproteinase 2 (MMP2), MMP9 and MT1-MMP to digest the extracellular matrix and are able to degrade matrix to a greater extent than podosomes (2, 20). Invadopodia are typically greater than 2 µm in length, have a lifespan of hours and less than 10 are usually present on a cell (1). The rigidity of the extracellular matrix is an important determinant of invadapodia formation with more rigid matrices promoting their formation (21). Invadopodia appear as puncta with small clusters of a few large actin-rich dots (15).

### Molecular features

Many proteins, including tyrosine kinases, proteases and adapter proteins are involved in the assembly and maturation of invasive projections. **Figure 1** depicts the structure of invasive projections of a cell with specific structural and common molecular features of a podosome and an invadopodia. Many proteins have been found in, or associated with, both structures. As described in **Table 1**, while many studies have focused on determining components that are unique to either podosomes or invadopodia in various cell types, in order to definitively identify these structures, to date, no specific marker has been identified that distinguishes invadopodia from podosomes. Despite this, some distinct patterns of protein localization have been demonstrated. The presence of vinculin has been proposed to be a marker for a podosome (5) while the localization patterns of the adaptor proteins Nck1 and Grb2 have been reported to distinguish invadopodia from podosomes (22).

**Table 1:** Depicts protein characterization identified in podosomes and invadopodia in various cell types.

Feature/Protein	Description	Cell Type Studied	Found In	Reference
Cortactin	Actin-binding protein involved in cytoskeletal organization	MDA-MB-231 breast cancer cells	Invadopodia	(70)
N-WASP	Regulates actin polymerization and cytoskeletal dynamics	MDA-MB-231 breast cancer cells	Invadopodia	(71)
Arp2/3 Complex	Actin nucleation factor that promotes branching	MDA-MB-231 breast cancer cells	Both podosomes and invadopodia	(72)
TKS5	Scaffolding protein essential for invadopodia formation	MDA-MB-231 breast cancer cells	Both podosomes and invadopodia	(73)
MT1-MMP	Matrix metalloproteinase involved in ECM degradation	MDA-MB-231 breast cancer cells	Invadopodia	(74)
Fascin	Actin-bundling protein associated with invasive potential	MDA-MB-231 breast cancer cells	Invadopodia	(75)
Integrins (e.g., β1, αV)	Cell adhesion receptors mediating ECM interactions	Endothelial cells	Both podosomes and invadopodia	(76)
Paxillin	Focal adhesion protein involved in signal transduction	Osteoclasts	Podosomes	(77)
Vinculin	Actin-binding protein that stabilizes cell adhesion	Osteoclasts	Podosomes	(78)
Talin	Links integrins to the actin cytoskeleton	Osteoclasts	Podosomes	(79)
MMP-2	Matrix metalloproteinase involved in ECM remodeling	Trabecular meshwork cells	Podosomes	(80)
MMP-14	Matrix metalloproteinase critical for ECM degradation	Trabecular meshwork cells	Both podosomes and invadopodia	(81)
Cdc42	Small GTPase regulating actin cytoskeleton	MDA-MB-231 breast cancer cells	Invadopodia	(82)
Caldesmon	Actin-binding protein regulating actomyosin interactions	A7r5 aortic smooth muscle cells	Podosomes	(83)
α-Actinin	Actin cross-linking protein stabilizing cytoskeletal structures	Osteoclasts, MDA-MB-231 breast cancer cells	Both podosomes and invadopodia	(84)
Fibronectin	ECM glycoprotein involved in cell adhesion and migration	Trabecular meshwork cells	Podosomes	(85)
Versican	ECM proteoglycan involved in cell adhesion and proliferation	Trabecular meshwork cells	Podosomes	(86)
WASP	Actin nucleation-promoting factor	MDA-MB-231 breast cancer cells	Both podosomes and invadopodia	(87)
Tyrosine Kinase Substrate	Adapter protein involved in invadopodia signaling	MDA-MB-231 breast cancer cells	Both podosomes and invadopodia	(88)
Actin Filaments	Structural filaments forming the cytoskeleton	Osteoclasts, MDA-MB-231 breast cancer cells	Both podosomes and invadopodia	(89)
Src Kinase	Tyrosine kinase involved in invadopodia formation	MDA-MB-231 breast cancer cells	Invadopodia	(90)
Dynamin	GTPase involved in vesicle trafficking and invadopodia turnover	MDA-MB-231 breast cancer cells	Invadopodia	(91)
β-Actin	Cytoskeletal protein essential for invadopodia structure	Fibrosarcoma	Both podosomes and invadopodia	(92)
Filamin A	Actin-binding protein that cross-links filaments	MCF-7-ErbB2, MDA-231, MDA-231-ErbB2, and BT-20 Breast cancer cells	Invadopodia	(93)

Key proteins in the formation of both podosomes and invadopodia are those associated with actin nucleation, actin binding, kinases and scaffold proteins that regulate the actin organization within the structures (2, 23). Podosomes are rich in F-actin (localized to their core) and proteins that regulate the actin cytoskeleton assembly and disassembly. These include actin related protein 2/3 complex (ARP2/3), cortactin, gelsolin, Neural Wiskott-Aldrich syndrome protein (N-WASP) and WASP-interacting protein (WIP). Invadopodia are also rich in actin filaments and proteins that regulate components of the actin cytoskeleton. In the initial stages, tyrosine kinase substrate with five SH3 domains (TKS5) co-localizes with cortactin in precursors of invadosomes, suggesting that TKS5 recruits cortactin in a crucial initiation step of formation (24). TKS5 also recruits and interacts with many other proteins both directly and indirectly, including growth factor receptor-bound protein 2 (GRB2) and the actin regulators Nck1, Nck2 and N-WASP (25). Cortactin is also associated with and/or regulated by many other proteins. These include the ARP2/3 complex, WASP-interacting protein (WIPF1), and dynamin. All of these are key players in the formation of both podosomes and invadopodia (5). Cortactin can be phosphorylated by PAK1, regulating its interactions with the aforementioned proteins (15). The co-localization of cortactin with phosphotyrosine is a key marker of matrix-degrading invadopodia (26). In regards to matrix degradation, TKS4 has been shown to regulate the localization of MMPs, while cortactin controls the secretion of these proteases (21, 23, 27).

While they share some proteins, the way these proteins function may differ between podosomes and invadopodia. For example, it has been shown that invadopodia formation is dependent on the actin cytoskeleton and does not require microtubules or vimentin intermediate filaments (28). Podosome function on the other hand has been found to require intact microtubules (1, 29).

As well as proteins, the membranes that surround podosomes and invadopodia have differing lipid composition. Specifically, podosomes and invadopodia differ in their predominant phosphatidylinositol (PI) phosphate composition. Podosomes are rich in phosphatidylinositol-3,4,5-triphosphate (PtdIns(3,4,5)P3), while invadopodia exhibit a higher abundance of phosphatidylinositol-3,4-bisphosphate (PtdIns(3,4)P2) and phosphatidylinositol-3-phosphate (PtdIns(3)P) (2, 30). These lipids play a key role in the recruitment and activation of the signaling intermediates which are localized to invasive projections (30). Furthermore, invadopodia formation has been demonstrated to be dependent on the availability of cholesterol, with invadopodia having the properties of cholesterol-rich lipid rafts (31).

The matrix-associated surface of podosomes is rich in integrins which anchor the structure to the extracellular matrix (6). There are two main classes of proteases present in podosomes and invadopodia that facilitate ECM degradation (27). These include zinc-regulated metalloproteinases MMP2, MMP9 and MT1MMP (21), and the ADAM (a disintigrin and metalloproteinase) family, including ADAM12, ADAM 15 and ADAM19 (1). MMPs as well as the ADAM family are markers of a highly invasive phenotype and are highly expressed in invadopodia-forming cells (32).

The formation of invasive projections has been shown to be dependent on the activity of several different signaling pathways. Formation of invadopodia has been shown to depend on the activity of the tyrosine kinases Tsk5 (33) and c-Abl (34). The adaptor protein Tks5, a substrate for Src kinase, localizes to invadopodia and has been shown to be necessary for invadopodia formation and invasive behavior in several cancer cell lines (33). Abl kinase has recently been shown to be an essential regulator of invadopodia assembly and function (34).

## Novel models of cell invasion

It was initially hypothesized that cell type determined which structures were produced, with podosomes exclusive to normally invasive cells (i.e. macrophages, endothelial cells, smooth muscle cells), and invadopodia only formed by highly invasive carcinoma cells. However, a study showed that podosomes were formed by non-invasive oral squamous cell carcinoma (SCC) cells, but after these cells underwent epithelial-mesenchymal transition and became invasive, they began to produce invadopodia (35). These podosomes that were found on non-invasive SCC cells were atypical from classically described podosomes, in that they all formed *de novo*, where usually new podosomes form by fragmenting off pre-existing podosomes. They also had a longer lasting life span, with life span being more comparable to that of invadopodia (35).

Another example of podosomes behaving similarly to invadopodia is seen in osteoclasts during bone remodeling. During this process, osteoclasts turn from a migratory phenotype to one with resorptive activity, remaining stationary where sites of bone-matrix degradation are situated. It is well documented that MMP14 localizes to both podosomes and invadopodia, and it had recently been found that MMP14 also localizes to osteoclast podosomes, but the mechanism used is similar to that of invadopodia (36). In light of these findings, it has been suggested that podosomes may be a precursor structure to invadopodia, and it is only when it is required that invadopodia form (36). Following from this, it was also suggested that invadopodia represent a physiological form of podosomes that are actively associated with the localized degradation of the matrix.

Another suggestion is that cells may have an innate ability to form both podosomes and invadopodia, and the formation of one or the other may be dependent on the matrix the cells are situated on, as well as the signaling pathways activated at the time (35). Previous studies demonstrated the presence of invadopodia and focal adhesions in both 2D and 3D *in vitro* environments (37–39). Normally, physiological migration and invasion of cells is tightly regulated, and if the formation of invadopodia requires specific signaling pathways, formation will not occur if culture conditions are incorrect.

### Trophoblast cell invasion

A critical event early in human pregnancy is the transformation of the uterine spiral arterioles to create a high flow, low resistance vasculature. Successful remodeling of the maternal uterine vasculature facilitates increased maternal blood flow to the placenta thus ensuring normal fetal growth and development (40). This requires invasion of trophoblast cells which move from the placenta into the maternal uterine vessels and surrounding matrix and integrate into the vessel walls (41, 42).

**Figure 2:**
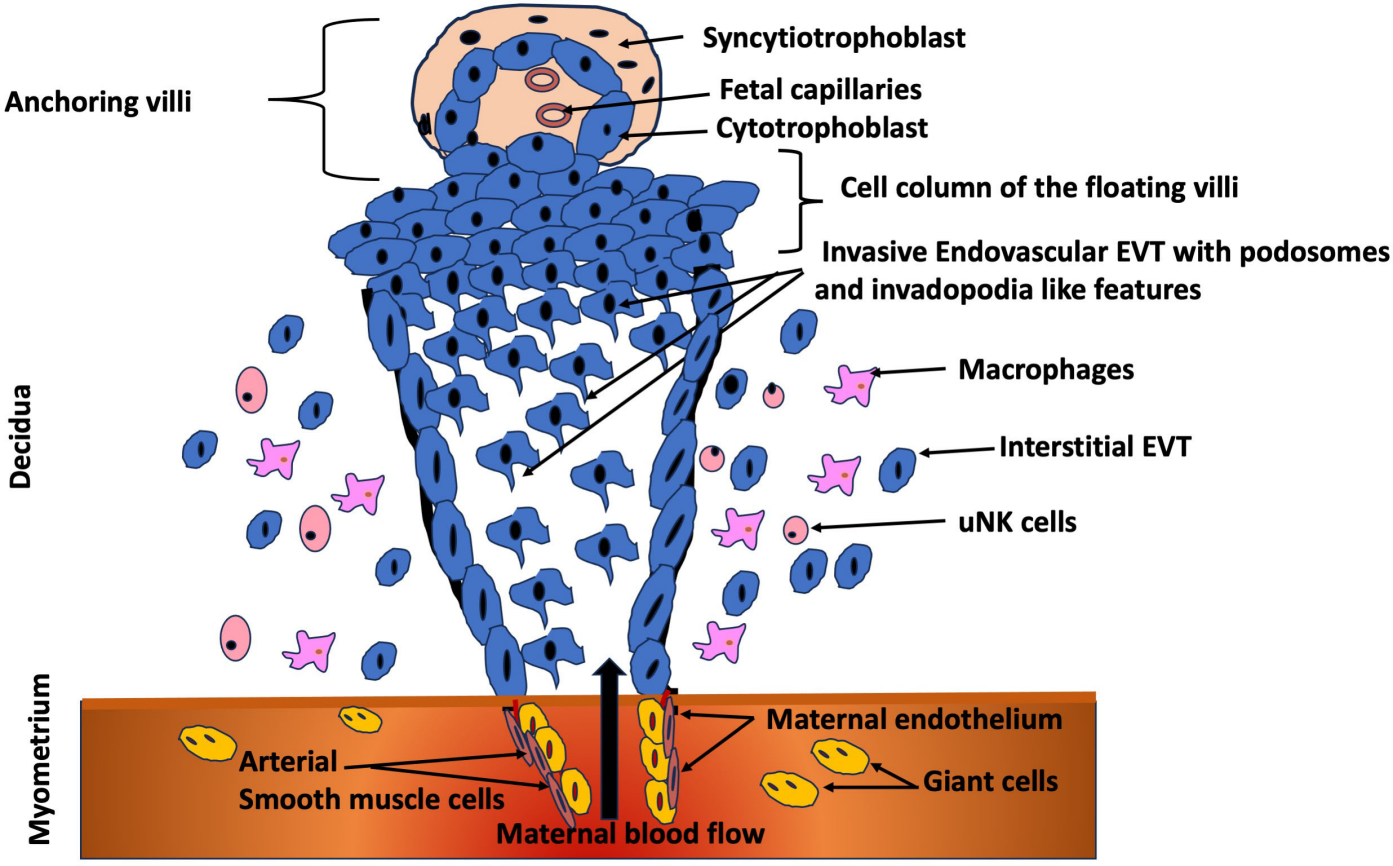
Trophoblast invasion at the maternal-fetal interface. Extravillous trophoblasts (EVT) originating from the trophoblastic shell of the anchoring villi proliferate in the cell column, migrate to the decidua and subsequently invade the myometrial stroma (41), by displaying podosomes and invadopodia-like features. Subsequently, invade the lumen of the arterioles to replace the endothelium of the maternal vessels (94). Figure adapted and modified from Lunghi et al. (2007) (95).

As depicted in **Figure 2**, the trophoblast cells that carry out the remodeling process are characterized by a gain of invasive ability. This phenotypic change enables them to move away from the tips of the placental villi and migrate into the maternal tissue (43–45). In order to become invasive, a subset of trophoblast cells must begin to secrete matrix metalloproteinases (MMPs) (44, 45). The ability to express the gelatinases MMP-2, MMP-9 and MMP-12 (46, 47) confers these trophoblast cells with the capacity to degrade elastin, collagens and laminin, thus enabling them to invade through the extracellular matrix of the uterine decidual stroma and integrate into the walls of the spiral arterioles. The remodeling process occurs in a very defined time frame and space, commencing early in the first trimester of pregnancy and continuing into the second trimester before ceasing at around 16–20 weeks of gestation with cells invading only as deep as the first third of the myometriumm (42, 45, 48).

As depicted in **Table 2**, the invasion of trophoblast cells during human pregnancy is often considered analagous to the invasion of malignant cancer cells as both are highly invasive, the adhesion molecules and proteases involved are similar and the cells use similar strategies to evade the host immune system (49). However, the major difference is that trophoblast cell invasion is very tightly controlled and regulated by the complex interplay of growth factors, cytokines, endocrine factors, oxygen concentrations and haemodynamics at the maternal-fetal interface. These act both temporally and spatially to initially promote and then limit the extent of trophoblast cell invasion (43, 50). While the similarities between invasive trophoblast cells and cancerous cells have been described, particularly their ability to digest matrix via secretion of MMPs, only one recent study (51) has examined whether trophoblast cells form podosomes similar to other non-cancerous cells or whether they form invasive projections that are invadopodia-like. The evidence suggests that trophoblast cells form atypical invasive projections that are neither podosomes or invadopodia but that exhibit features of both structures (51). Furthermore, the focal adhesion molecules that are mission control for trophoblast invasion, regulates adhesion, signaling, cytoskeletal changes, and ECM interaction to ensure that invasion is precise, regulated, and adaptive (52–54). Focal adhesion molecules also allow trophoblasts to “sense” mechanical cues, oxygen levels, and ECM stiffness that are essential for them adapt to their invasion depth. In low oxygen, for example, certain pathways (e.g. hypoxia inducible factor-1, HIF-1α) are triggered, modulating focal adhesion signaling and promoting invasion early in pregnancy (55). Unlike cancer cells, trophoblasts need to attach just enough to anchor maternal decidua, but also stay mobile enough to invade. Focal adhesion dynamics control this balance through turnover and recycling of adhesions.

**Table 2:** Invasive projections of trophoblasts and cancer cells.

Feature	Trophoblast Projections	Cancer Cell Projections
Types of projections	Podosomes, invadopodia-like structures	Invadopodia, filopodia (thin, exploratory projections), lamellipodia (broad ruffling edges), microtentacles for migration and invasion.
Function	Penetrate maternal penetrate the decidua and remodel spiral arteries to establish a physiological placental interface	Degrade ECM for invasion and metastasis of aggressive cancer
Actin Organization	Organized, often podosome-like (actin core with adhesion ring)	Invadopodia: actin-rich core, MMP-rich zone
MMP Expression	Regulated expression of MMP-2, MMP-9	Over expression of MMP-2, MMP-9, MT1-MMP
Stability	Transient, tightly regulated	Longer-lived, more stable in invasive cells
Regulation	Hormonal (e.g., hCG), cytokines, maternal signals including modulation by maternal immune cells	Oncogenes (e.g., Src, Ras), hypoxia, inflammation
Polarity	Directed, guided invasion	Multidirectional and unguided invasion
Outcome	Controlled invasion for placental anchoring and nutrient exchange for physiological adaptation	Uncontrolled invasion leading to metastasis.

Freshly isolated primary first trimester trophoblast cells grown on Matrigel for 48 hours to promote acquisition of the invasive phenotype leads to expression of mRNA for key proteolytic enzymes, known to be associated with invadopodia. Expression of mRNAs for the gelatinases MMP2, MMP9, MT1-MMP (MMP14 precursor) and MMP14 have all been detected (56). These enzymes are secreted by mature invadopodia and enable them to digest extracellular matrix to a greater extent than podosomes. In addition, expression of mRNAs for the membrane bound metalloproteinases ADAM8, ADAM12, ADAM15 and ADAM19 can also be detected (57). While a role for ADAMs in invadopodia is less well defined, ADAM12, ADAM15 and ADAM19 have been shown to interact with the Tks5 adaptor protein which localizes to invadopodia and ADAM8 has also been detected in podosomes (51). This suggests that components associated with invadopodia rather than podosomes are present in first trimester trophoblast.

## Invasive projections as therapeutic targets

Invasive projections represent a new and exciting therapeutic target. Recent studies highlight the proteomic and transcriptomic profiling of invasive cells, with an emphasis on proteins associated with invadopodia and their structures (58) (59). Inhibition or stimulation of invasive projection formation can be the basis for disease therapy where cell invasion is abnormal. Potentially, invasive projections present two paths that can be exploited in the therapeutic arena. First, matrix degradation *in vitro* by invasive projections can, and is, being used to test the effect of lead compounds and to screen drug libraries (60). Secondly, invasive projections can be a target for therapy where disruption or stimulation of their formation is beneficial. The development of novel therapies is hindered by a lack of knowledge of podosome or invadopodia-specific components. Thus, there is an urgent need to define the composition of invasive projections on cells of differing origins and invasive potentials (14).

### Over-invasion

In cancer biology, inhibition of invadopodia formation has been proposed as a strategy for treating breast, lung and pancreatic cancers (2, 61–63). This approach is considered appealing as cell viability is not affected by targeting invadopodia thus therapies based on this strategy would be anticipated to have fewer side effects than current therapies (64). In addition, a therapy targeting invadopodia formation will only target the cancer cell population thus vastly improving drug selectivity (65).

Abnormal trophoblast cell invasion is associated with very serious pregnancy complications that can be life-threatening for the mother and have serious health consequences for the baby which is often born premature. Over invasion results in complications including placenta percreta, increta and accreta, where cells invade into maternal tissues beyond the uterine wall (66), and gestational trophoblastic diseases such as choriocarcinoma and invasive mole with highly invasive, metastatic tumors (67). It could be envisaged that a therapy targeting a unique property of the invasive projections formed by trophoblast cells could be employed to treat these conditions.

### Under-invasion

Switching cell invasion on could also be beneficial in some instances. Transient activation of invasive projection formation has been suggested as potentially useful in the treatment of skeletal anomalies, where mutations in podosome proteins cause several distinct craniofacial defects (2, 68). Several serious pregnancy complications are characterized by under invasion of trophoblast cells. Shallow trophoblast cell invasion is associated with pre-term delivery, pre-eclampsia and fetal growth restriction (41, 45), and possibly some instances of miscarriage (69). These represent pathologies where there are currently no effective therapies other than delivery of the baby which is undesirable pre-term. Just as for pregnancy complications with over-invasive cells, targeting the invasive projections formed by trophoblast cells could be a new and novel therapeutic strategy.

## Conclusions

Though sharing similarities with cancer cell invasion, trophoblast cell invasion is highly regulated compared to the dysregulated invasion seen in cancer. Trophoblast cells form invasive structures with unique properties (51) but little is known about their molecular characteristics or regulation. Evidence suggests the structures formed by trophoblasts are atypical, being neither a true podosome nor invadopodia. Comparing trophoblast cell and cancer cell invasion, as well as other novel models of cell invasion, will contribute to a better understanding of the classification and regulation of podosomes and invadopodia, which will ultimately inform and improve disease treatment strategies that aim to modify cell invasion. The identification of the similarities and differences could provide novel targets for the diagnosis and treatment of pathological pregnancies, cancer and other diseases where cell invasion is abnormal, enabling the translation of basic research discoveries into clinical applications.
